# Light Therapy Alleviates Addiction‐Related Symptoms and Reshapes Habenula and Midbrain Pathways

**DOI:** 10.1002/advs.202514044

**Published:** 2026-01-14

**Authors:** Jinhui Li, Huiyang Huang, Cunhao Hu, Xuan Zhang, Song Lin, Xiaodan Huang, Ti‐fei Yuan, Kwok‐Fai So, Lu Huang, Chaoran Ren, Qian Tao

**Affiliations:** ^1^ Division of Medical Psychology and Behavior Science School of Medicine Jinan University Guangzhou China; ^2^ Physiology Department School of Medicine Jinan University Guangzhou China; ^3^ Key Laboratory of CNS Regeneration (Ministry of Education) Guangdong Key Laboratory of Non‐human Primate Research GHM Institute of CNS Regeneration Jinan University Guangzhou China; ^4^ Shanghai Key Laboratory of Psychotic Disorders Brain Health Institute National Center for Mental Disorders Shanghai Mental Health Center Shanghai Jiaotong University, School of Medicine Shanghai China; ^5^ Department of Rehabilitation Medicine First Affiliated Hospital of Jinan University Guangzhou China

**Keywords:** dorsal raphe nuclei, habenula, internet gaming disorder, light therapy, orbitofrontal cortex, ventral tegmental area

## Abstract

The treatment of internet gaming disorder (IGD) poses a significant challenge. Light therapy has demonstrated efficacy in preventing cocaine‐driven reinstatement in a rodent model. The potential of light therapy, along with its underlying neural mechanisms, in treating IGD remains largely uninvestigated. A total of 104 participants are included in the RCT and 69 in the fMRI analysis. The intervention involves 14 daily sessions of light therapy, light placebo, or cognitive training. At post‐intervention, light therapy group shows treatment effects comparable to those of cognitive training group, with greater reductions in addiction severity, weekly gaming duration, craving, and withdrawal symptoms than the placebo group. Longitudinal fMRI shows reduced activation in right inferior orbitofrontal cortex (iOFC), left inferior frontal gyrus, and right insula. Using habenula, ventral tegmental area (VTA), and dorsal raphe nuclei (DRN) as seeds, light therapy increases functional connectivity between habenula and medial OFC (mOFC), and VTA and mOFC. These increases are significantly correlated with reductions in withdrawal/craving symptoms. Importantly, changes in withdrawal symptoms mediate the association between habenula‐mOFC functional connectivity and addiction severity. Our findings suggest light therapy is effective in alleviating withdrawal symptoms and ultimately reducing addiction severity by reshaping the habenula‐mOFC functional connectivity in IGD.

## Graphical abstract

This study investigates light therapy for treating Internet Gaming Disorder (IGD). Functional MRI (fMRI) data reveal that light therapy enhances brain connectivity, particularly between the habenula and the medial orbitofrontal cortex (mOFC). These changes are associated with reduced withdrawal symptoms and addiction severity, highlighting the potential of light therapy as a novel therapeutic approach for IGD.

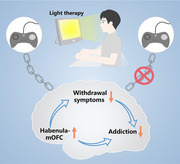

## 1 Introduction

In the digital era, internet gaming has emerged as a popular form of entertainment, with an estimated 2.5 billion online gamers worldwide [1]. While most individuals enjoy internet gaming without adverse effects, some may develop mental health symptoms. Recognized as a psychiatric diagnosis, internet gaming disorder (IGD) has been included in both the Diagnostic and Statistical Manual of Mental Disorders, 5th edition (DSM‐5) [2] and International Classification of Diseases, 11th revision (ICD‐11) [3]. IGD is associated with significant impairments and negative consequences, such as academic or occupational decline, impaired interpersonal relationships, and reduced life satisfaction [4]. Current treatments for IGD include medication, cognitive‐behavioral intervention, and others [5]. However, existing evidence supporting medications remains limited, with no medications have formal indications for IGD [6]. With regards to cognitive‐based interventions, IGD individuals frequently demonstrate poor attendance, reluctance to engage in therapeutic processes, and high dropouts [6]. Although research on IGD interventions is steadily accumulating, there remains a critical need for more accessible, cost‐effective, and patient‐friendly therapeutic options to address this challenging disorder [5].

Light therapy, a safe and cost‐effective nonpharmacological intervention, has been widely applied in clinical and research settings for various psychiatric conditions, including depression [7, 8], sleep disorders [9], cognitive disorders [10], and pain [11]. Our recent work reported that bright light treatment can prevent cocaine‐driven relapse and improve aversive emotional states associated with drug withdrawal [12]. Indeed, IGD and other forms of addiction (such as substance use disorder, SUD) share core pathological and symptom characteristics. For instance, both IGD and SUD demonstrate intense cravings for specific behaviors or substances during the initial abstinence period. The craving then triggers significant withdrawal symptoms, such as negative emotions [13]. Crucially, such withdrawal responses are strongly associated with high relapse rates, posing a significant challenge for addiction recovery [12]. Accumulating evidence suggests that light therapy is effective in reducing negative emotions [14], highlighting its significant role as a promising strategy for treating addiction disorders, including the case of IGD.

Furthermore, our animal study identified the lateral habenula neural pathway as a mediator of the therapeutic effects of bright light treatment for SUD [12]. The habenula and its related circuitry are highly conserved across species [15]. A series of our previous works have shown that habenula circuits play a crucial role in the effects of bright light therapy in a rat depressive model [7, 8], a stress‐induced sleep model in rats [16], and individuals with subthreshold depression [17]. Within the context of addiction, the habenula is particularly significant due to its involvement in negative affect, reward processing, cognitive control, and stress [18, 19]. Impairments in these functions are consistently observed during addiction abstinence [20]. The habenula neurons project to the ventral tegmental area (VTA) and the dorsal raphe nuclei (DRN), two midbrain nuclei critically involved in addiction through widespread release of dopamine and serotonin [21, 22]. Importantly, habenula, VTA, and DRN have consistently demonstrated modulation effects by light [14, 17]. This suggests they play a crucial role in the neural mechanisms underlying the efficacy of light therapy for IGD and represent promising therapeutic targets for addiction treatment [22, 23].

Light therapy, owing to its convenience, ease of use, safety, few side effects, and patient‐friendliness, shows significant potential for widespread applications in addiction intervention, especially for IGD. However, there is currently a lack of empirical evidence and randomized controlled trial (RCT) supporting its efficacy. To our knowledge, this is the first clinical study to examine the efficacy of light therapy for IGD. Additionally, we employed a craving task‐based functional magnetic resonance imaging (fMRI) approach to investigate the neural mechanisms underlying light therapy. We hypothesized that light therapy would alleviate addiction‐related symptoms, including IGD severity, weekly gaming duration, withdrawal, and craving symptoms, and alter functional connectivity of habenula, VTA, and DRN.

## 2 Results

### 2.1 Participants

**FIGURE 1:**
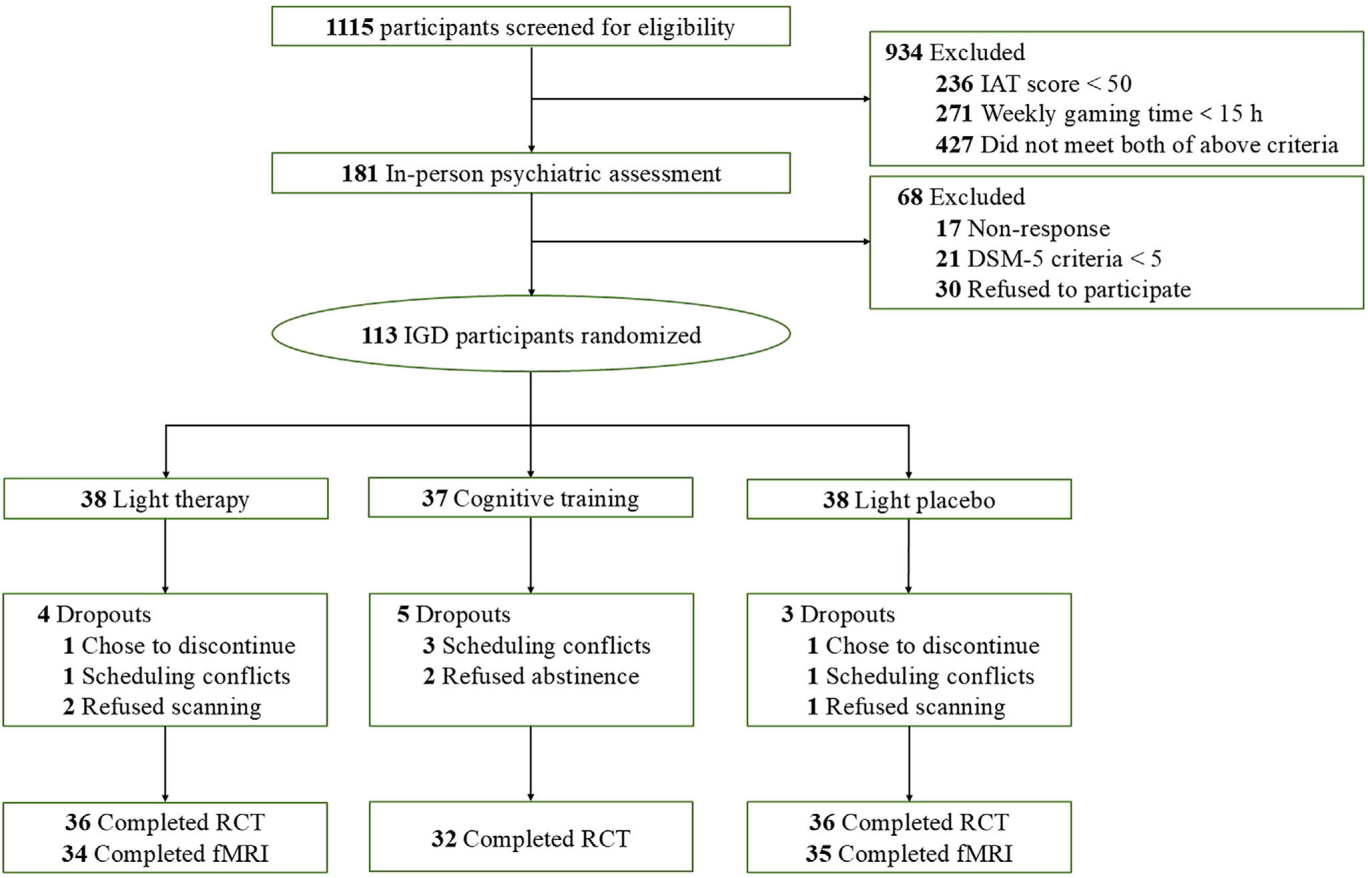
CONSORT flow diagram. Abbreviations: IGD, internet gaming disorder; IAT, internet addiction test; DSM‐5, Diagnostic and Statistical Manual of Mental Disorders, 5th edition; fMRI, functional magnetic resonance imaging; RCT, randomized clinical trial.

The recruitment process is shown in Figure 1. For the RCT study, a total of 113 eligible participants were randomized and 9 participants did not complete the intervention, resulting in 104 participants completed the RCT study (light therapy *n* = 36, light placebo *n* = 36, cognitive training *n* = 32). The participants in the light therapy and light placebo groups were also invited to participate the fMRI study, and 3 declined the fMRI scan, resulting in 69 completed the fMRI study (light therapy *n* = 34, light placebo *n* = 35). There were no significant between‐group differences in baseline demographic or outcome measures (Table 1). The mean (SD) age was 20.56 (2.08) years, and 45 (43%) participants were female. Overall, 93% of participants reported being satisfied with the light intervention, 61% found it convenient, and 75% stated they would recommend the light intervention to a friend.

**TABLE 1:** Demographic and clinical characteristics of participants.

Characteristics	Light therapy (*n *= 36)	Light placebo (*n *= 36)	Cognitive training(*n *= 32)		
	Mean (SD)	Mean (SD)	Mean (SD)	*F*	*P*
Age (years)	20.1 (1.8)	20.7 (2.3)	20.8 (2.1)	1.15	0.32
DSM‐5 criteria	6.8 (1.3)	6.6 (1.5)	6.7 (1.5)	0.34	0.71
IAT	65.4 (10.5)	64.3 (8.9)	67.1 (9.7)	0.74	0.48
Weekly gaming time (hours)	23.8 (7.2)	22.2 (5.7)	22.5 (6.1)	0.67	0.51
BAI	8.5 (6.3)	6.3 (6.0)	6.9 (6.5)	1.18	0.31
BDI	13.4 (7.5)	12.7 (9.0)	12.8 (7.6)	0.07	0.93
PSQI	7.0 (3.1)	7.0 (2.7)	7.1 (2.8)	0.01	0.99
	N (%)	N (%)	N (%)	*χ^2^*	*P*
Female	14 (39)	17 (47)	14 (44)	0.51	0.77
Education				0.23	0.32
Undergraduate	32 (89)	30 (83)	24 (75)		
Graduate	4 (11)	6 (17)	8 (25)		
Type of game				5.79	0.21
Mobile game	23 (64)	23 (64)	22 (69)		
Personal computer game	10 (28)	4 (11)	6 (19)		
Both	3 (8)	9 (25)	4 (12)		
Alcohol using	4 (11)	3 (8)	2 (6)	0.57	0.91
Tobacco smoking	1 (3)	0 (0)	0 (0)	1.77	1.00

Abbreviations: DSM‐5, Diagnostic and Statistical Manual of Mental Disorders, 5th edition; IAT, Young's Online Internet Addiction Test; BAI, Beck Anxiety Inventory; BDI, Beck Depression Inventory; PSQI, Pittsburgh Sleep Quality Index.

### 2.2 Outcomes

**FIGURE 2:**
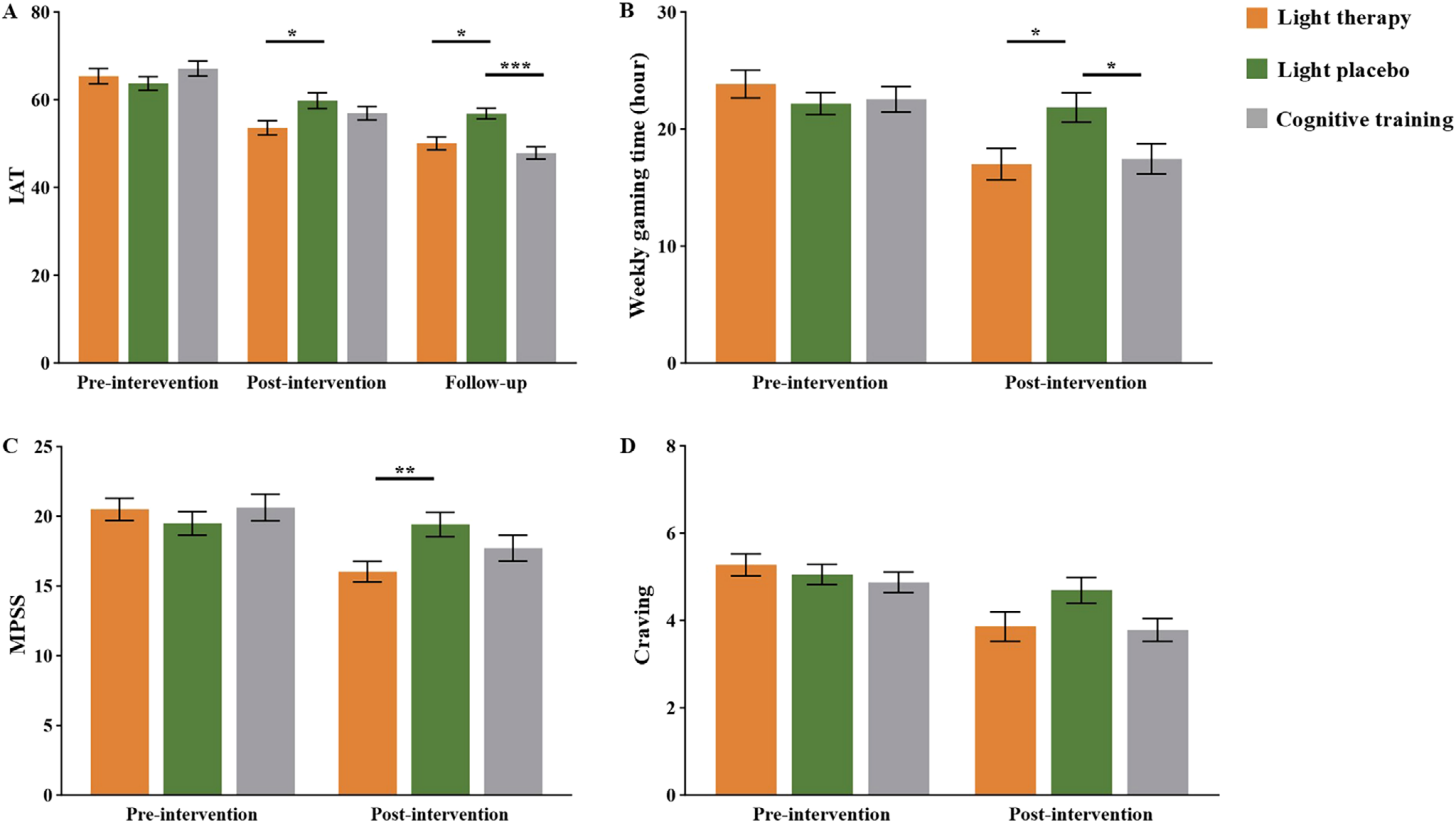
Outcome assessment. Primary and secondary outcome assessments. (A) Addiction severity, assessed with IAT, at pre‐intervention, post‐intervention, and follow‐up; (B) to (D), Pre‐intervention and post‐intervention assessments of (B) weekly gaming time, recorded through game history or game‐tracking software; (C) withdrawal symptoms, assessed with MPSS; (D) craving symptoms, assessed with two items of MPSS. At pre‐intervention, there were no significant differences among the three groups in addiction severity, weekly gaming time, withdrawal, or craving symptoms. In contrast, there were significant differences in addiction severity, weekly gaming time, withdrawal, and craving among the three groups at post‐intervention. Moreover, there was a significant between‐group difference in addiction severity at follow‐up. Abbreviations: IAT, Internet Addiction Test; MPSS, mood and physical symptoms scale; Error bars indicate standard error. ^*^ *p* < 0.05, ^**^ *p* < 0.01, ^***^ *p* < 0.001.

The mean (SD) changes of Young's Internet Addiction Test (IAT) score from baseline to post‐intervention were 11.81 (9.71) in the light therapy group, 3.92 (7.98) in the light placebo group, and 10.16 (9.62) in the cognitive training group. There were significant group‐by‐time interactions for IAT score (*F*_201.36_ = 9.66, *p *< 0.01). At post‐intervention, the preplanned simple contrasts found significant effects of light therapy vs. light placebo (*p *= 0.02), but not for light therapy vs. cognitive training (*p *= 0.41) and cognitive training vs. light placebo (*p *= 0.40) (Figure 2A). The effect sizes were 0.92 (95%CI, 0.22–1.61) for light therapy vs. light placebo, 0.46 (95%CI, −0.25 to 1.17) for light therapy vs. cognitive training, and 0.46 (95%CI, −0.25 to 1.16) for cognitive training vs. light placebo. At follow‐up, there were significant effects of light therapy vs. light placebo (*p *= 0.01) and cognitive training vs. light placebo (*p *< 0.001), but not for light therapy vs. cognitive training (*p *= 0.47) (Figure 2A). The effect sizes were 0.98 (95%CI, 0.28–1.68) for light therapy vs. light placebo, 0.42 (95%CI, −0.29 to 1.13) for light therapy vs. cognitive training, and 1.40 (95%CI, 0.67–2.14) for cognitive training vs. light placebo.

The results also revealed significant interactions for weekly gaming time (*F*_99.83_ = 8.80, *p *< 0.01), withdrawal symptoms (*F*_101.00_ = 9.87, *p *< 0.01), and craving (*F*_101.00_ = 3.08, *p *= 0.05). As for weekly gaming time, the preplanned simple contrasts found significant improvements of light therapy group vs. light placebo group (5.00 [95%CI, 0.92 to 9.08], *p *= 0.01) and cognitive training group vs. light placebo group (4.45 [95%CI, 0.32–8.59], *p *= 0.03) (Figure 2B). As for withdrawal symptoms, there were significant improvements of light therapy group vs. the light placebo group (3.66 [95%CI, 0.81 to 6.50], *p *< 0.01) (Figure 2C). As for craving, the between‐group effects were almost significant (light therapy group vs. light placebo group, 0.81, [95%CI, −0.09 to 1.71], *p *= 0.09; cognitive training group vs. light placebo group, 0.90, [95%CI, −0.02 to 1.83], *p *= 0.06) (Figure 2D). There were no significant effects for light therapy group vs. cognitive training group (weekly gaming time, 0.55 [95%CI, −3.66 to 4.76], *p *= 0.95; withdrawal symptoms, 1.98 [95%CI, −0.95 to 4.91], *p *= 0.25; craving, 0.09, [95%CI, −1.03 to 0.84], *p *= 0.97), and cognitive training group vs. light placebo group (withdrawal symptoms, 1.68 [95%CI, −1.24 to 4.59], *p *= 0.36). There were no significant interactions for other indicators (Figure S1), including compulsive gaming behaviors (*p *= 0.29), compulsive gaming thoughts (*p *= 0.57), sleep quality (*p *= 0.79), and emotional regulation (*p *= 0.82).

### 2.3 Neuroimaging Results

**FIGURE 3:**
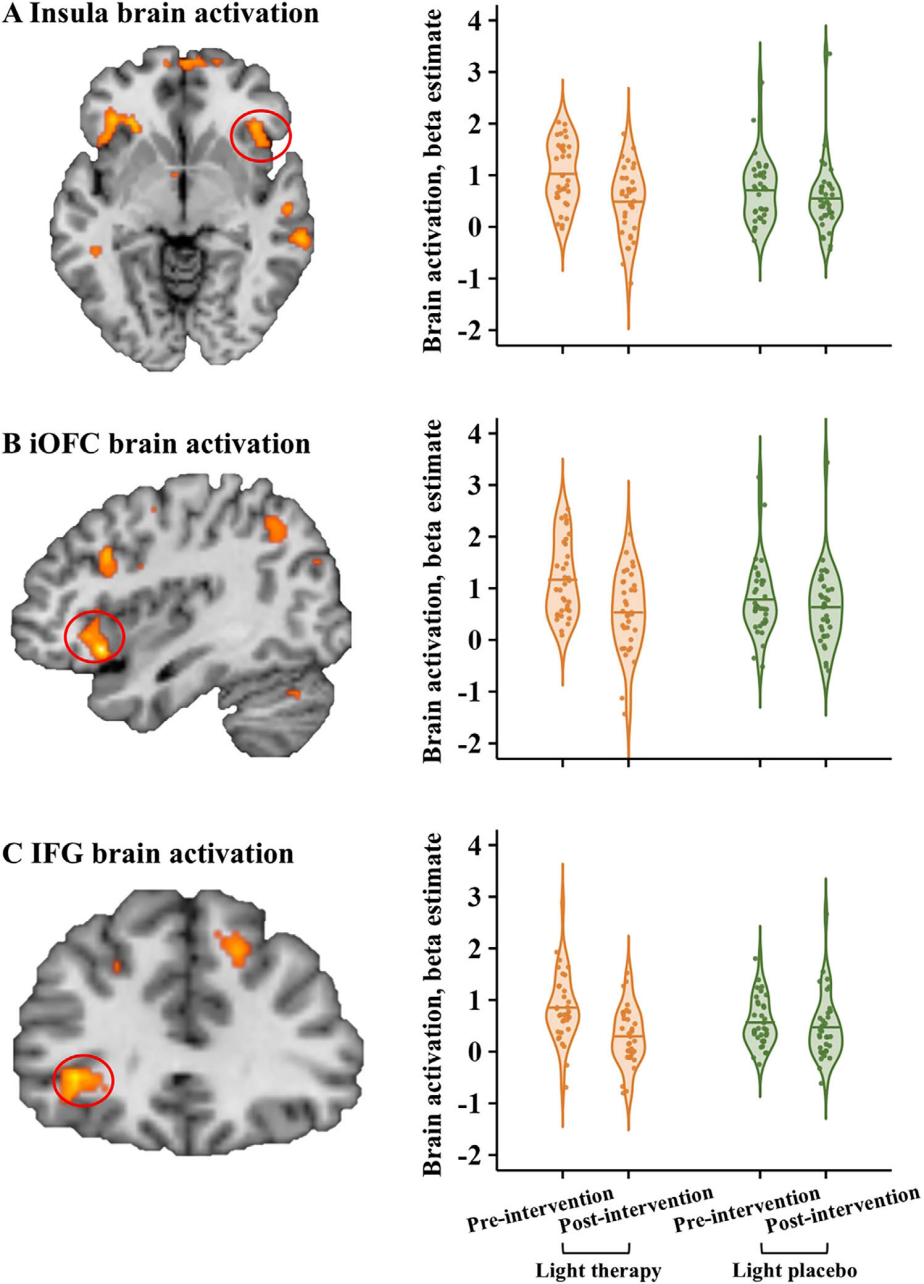
Intervention effects on neural responses during the craving task. Light therapy reduced brain activation in (A) right insula; (B) right iOFC; and (C) left IFG. The threshold was set at voxel‐level *p* < 0.001 and cluster‐level *P*_FWE_ < 0.05. Abbreviations: iOFC, inferior orbitofrontal cortex; IFG, inferior frontal gyrus; FWE, family‐wise error.

At baseline, all participants showed significant brain activations (gaming vs. typing) involving a widespread occipito‐temporal‐prefrontal‐cerebellar network, with no significant differences observed between the two groups (Table S1 and Figure S2). Whole‐brain ANOVA analysis (group × time) revealed significant interaction effects in right inferior and medial orbitofrontal cortex (iOFC, mOFC), bilateral insula, bilateral inferior frontal gyrus (IFG), right middle temporal gyrus (MTG), right medial superior frontal gyrus (mSFG), and left precentral gyrus (Table S2). At post‐intervention, light therapy group showed reduced activation in the right insula (Figure 3A), right iOFC (Figure 3B), and left IFG (Figure 3C) (all *t*_s_ ≥ 5.252, *P*_s_ < 0.001).

**FIGURE 4:**
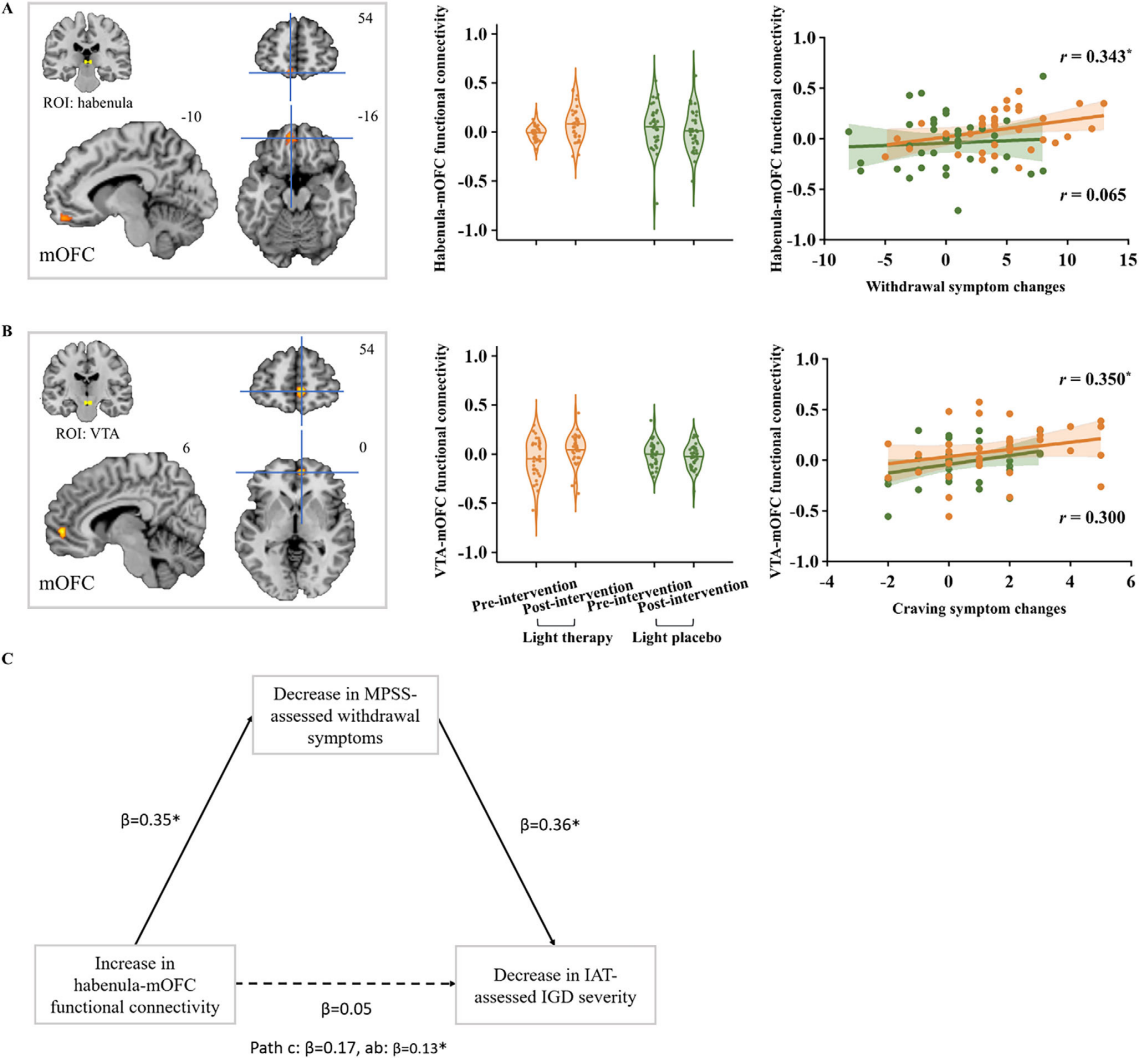
Intervention effects on functional connectivity during the craving task. (A) Bilateral habenula as the seed region, light therapy increased habenula‐mOFC functional connectivity from pre‐intervention to post‐intervention. The increased functional connectivity was correlated with reduced withdrawal symptom in the light therapy group (*p* = 0.047), but not in the light placebo group (*p* = 0.712). (B) Bilateral VTA as the seed region, light therapy increased VTA‐mOFC functional connectivity from pre‐intervention to post‐intervention. The increased functional connectivity was correlated with reduced craving symptom in the light therapy group (*p* = 0.042), but not in the light placebo group (*p* = 0.080). (C) The reduced withdrawal symptoms mediated the association between increased habenula‐OFC functional connectivity and decreased addiction severity (*b* = 6.10, [95% CI, 0.32–15.84], *β* = 0.13, *p* = 0.038). The threshold was set at voxel‐level *p* < 0.001 and cluster‐level *P*_FWE_ < 0.05. Abbreviations: VTA, ventral tegmental area; mOFC, medial orbitofrontal cortex; FWE, family‐wise error. Path c indicates the total effect; Path ab indicates the indirect effect.

Using the habenula/VTA/DRN as seed regions, whole brain ANOVA results revealed no significant interaction effects for the three ROIs at voxel‐level of *p* < 0.001 and cluster‐level family‐wise error (FWE) correction of *P*_FWE_ < 0.05. The results remained nonsignificant when using a less stringent threshold without FWE correction. Functional connectivity analyses revealed no significant between‐group differences at baseline. Using the habenula as ROI, the ANOVA analyses revealed a significant group × time interaction effect in mOFC and inferior temporal gyrus (ITG) (all *F*_s_ > 7.28). Using VTA as ROI, there was a significant interaction effect in mOFC (*F* > 4.76). Using DRN as ROI, there was a significant interaction effect in cerebellum, middle frontal gyrus (MFG), and precuneus (all *F*_s_ > 7.28) (Table S3). Post hoc analysis showed that compared with baseline, functional connectivity of habenula‐mOFC (Figure 4A) and VTA‐mOFC (Figure 4B) were significantly increased after the intervention in light therapy group (all *t*_s_ ≥ 2.049, all *P*_s_ < 0.05). Within the light therapy group, there were positive correlations between increased habenula‐mOFC functional connectivity and decreased withdrawal symptoms (*r *= 0.343, *p *= 0.047), and between increased VTA‐mOFC functional connectivity and decreased craving symptoms (*r *= 0.350, *p = *0.042). Additionally, the decrease in withdrawal symptoms was positively correlated with the decrease in IAT scores (*r *= 0.381, *p *= 0.026). In the mediation model, the pre‐post intervention changes in withdrawal symptoms mediated the association between changes in habenula‐mOFC functional connectivity and addiction severity (*b *= 6.10, [95%CI, 0.32–15.84], *β* = 0.13, *p *= 0.038) (Figure 4C). Approximately 73.55% of the effect of changes in habenula‐mOFC functional connectivity on changes in addiction severity was explained by changes in withdrawal symptoms. However, the direct effect of changes in habenula‐mOFC functional connectivity on changes in addiction severity was not significant (*b *= 2.19, [95%CI, −14.97 to 19.35], *β *= 0.05, *p *= 0.80). Application of the whole‐brain craving neuromarker revealed no significant group × time interaction effect (*F*_(1, 67)_ = 0.40, *p *= 0.53). The light therapy group showed no significant reduction in brain reactivity to craving (paired *t*‐test: *t*_33_ = 0.42, *p *= 0.68).

## 3 Discussion

The data reported herein represent the first clinical investigation into the effectiveness of light therapy for IGD and its underlying neural mechanisms. Compared to the placebo group, the light therapy group demonstrated significant treatment effects, showing greater reductions in IGD severity, weekly gaming time, withdrawal symptoms, and craving symptoms at post‐intervention. At the neural level, the light therapy group showed significantly reduced activations in insula, IFG, and OFC, and increased functional connectivity of habenula‐mOFC and VTA‐mOFC. These changes in functional connectivity were correlated with withdrawal or craving symptoms. Notably, withdrawal symptoms mediated the positive correlation between changes in habenula‐mOFC functional connectivity and IGD severity within the light therapy group.

There is a direct projection from OFC to habenula, and both are increasingly recognized as key neuroanatomical regions involved in regulating neural circuitry critical for addiction [24, 25]. The OFC‐habenula functional connectivity was positively correlated with compulsive drug‐taking despite receiving punishment [26]. Despite its small size in the human brain, numerous neuroimaging studies have successfully examined the habenula using both conventional [17, 27] and high‐resolution MRI techniques [28], demonstrating consistent effectiveness across resolutions. A recent human study using diffusion tractography demonstrated impaired habenula‐OFC structural connectivity in both short‐term abstainers and current cocaine users [27]. From a functional perspective, abnormal habenula activity has been reported in individuals with cocaine and heroin addiction [27] as well as nicotine addiction [29]. Furthermore, abnormal resting state functional connectivity between the habenula and OFC has been found in psychiatric individuals with SUD [30]. These findings, together with the present results, further highlight the crucial role of the habenula‐OFC pathway in IGD and its potential as a therapeutic target. In addition, the reduced craving is found to correlate with increased functional connectivity of VTA‐mOFC. This is consistent with previous findings suggesting that addictive cues elicit increased cravings, which have been reported to involve the VTA [31], and OFC [31, 32]. While impaired prefrontal control weakens the ability to suppress this cue‐elicited craving [33], cognitive regulation strategies have been shown to modulate craving through prefrontal systems exerting control on the VTA [34, 35].

Light therapy reduced activations in the insula, IFG, and OFC. Abnormalities in these brain regions have been reported among IGD individuals during exposure to gaming stimuli [36]. Abstinence‐induced cravings in smokers have been linked to increased cerebral blood flow in brain regions including the right OFC, right prefrontal cortex (PFC), and right insula [37]. Animal studies consistently suggest that inactivation of the insula leads to attenuation of drug seeking and intake, as well as the interruption of craving and addiction [38]. Similarly, human lesion studies have shown that damage to the insula is strongly predictive of smoking cessation [39]. In addition, many neuroimaging studies have revealed correlations between insula activity and craving ratings [37], underscoring its pivotal role in addiction‐related behaviors. In addition to the insula, neuroadaptations in OFC contribute to the core feature of addiction: impaired decision‐making [24, 40]. At the neuronal level, increased metabolism at OFC has been reported during early periods of abstinence in patients with cocaine dependence, with this hypermetabolism being positively correlated with craving [41]. In contrast, decreased involvement of OFC has been found in abstinent drug users during protracted withdrawal. These findings align well with a meta‐analysis study investigating OFC changes in patients with SUD [40].

Notably, addiction symptom improvements were correlated with functional connectivity changes, but not activation changes in specific brain regions. The neural mechanisms underlying behavioral improvements and light therapy may be more linked to integrated changes in communication efficiency at the brain network level. This perspective aligns with the “brain functional network” framework applied to psychiatric disorders [42]. While earlier research in psychiatric disorders primarily emphasized abnormalities within isolated brain regions, alterations in specific brain regions are not always correlated with changes in symptoms or behaviors. It is increasingly recognized that multiple brain regions act synergistically to support particular brain functions. In line with this view, growing evidence has suggested that dysfunction of brain functional networks is a hallmark of various psychiatric conditions [43], including addiction [44, 45]. Furthermore, intervention efficacy has been reported to correlate with functional connectivity changes in addiction patients. For instance, increased functional connectivity between the dorsal striatum and OFC was correlated with decreased gaming‐related emotional association bias in IGD individuals following cognitive training [8]. Similarly, in the context of light therapy, our previous clinical fMRI study revealed a significant correlation between increased VTA‐SFG functional connectivity and decreased depressive symptoms after treatment [17].

The current study establishes a long‐term bright light treatment paradigm for alleviating addiction symptoms in individuals with IGD. It is also noted that light has been used as an effective stimulus for reinforcing maladaptive behavior in the context of a conditioning paradigm. The above two phenomena are different in terms of lighting parameters, experiment context, and underlying circuitry. In the current light treatment paradigm, we used 14 daily continuous 30‐min bright white light illumination with a high intensity of 5000‐lux and demonstrated that habenula‐OFC functional connectivity was important for its efficacy. This light effect primarily relies on the non‐visual functioning of ipRGC and nonvisual circuits linking the retina to the brain. Within the context of the conditioning paradigm, brief light pulses (a few seconds) or chromatic light (such as 472 nm blue or 525 nm green light) are used as a type of sensory reinforcer [47, 48]. In addition to light cues, other stimuli can act as reinforcers, such as sound and novel objects [49, 50]. In this regard, light only acts as sensory stimuli, and the phenomenon may involve the visual circuits linking the retina and occipital cortex. The conditioning paradigms, relying on associative learning and rewarding memory, have been utilized to develop animal addiction models [51]. With relevance to IGD, a recent study developed an operant conditioning paradigm in mice using a brief light pulse to model digital technology‐based disorders and the underlying circuitry required further investigations [52].

The RCT results suggested that both light therapy and cognitive training were efficacious in reducing addiction, craving, and withdrawal symptoms compared with placebo. Cognitive training, a well‐established form of psychotherapy, serves as an active comparator in the current clinical trial. Regarding IGD, cognitive training primarily relies on association learning between addictive stimuli and negative emotions. Generally, cognitive training has significant limitations, such as being time‐consuming, requiring administration by professionals, and having uncertain long‐term effects. During training, participants must maintain attention on the training materials, which often leads to fatigue and increases the risk of dropout. Furthermore, attending cognitive training in psychiatric settings may heighten perceived stigma among patients [53]. In contrast, light therapy is a noninvasive physical intervention that offers several advantages. First, its adverse effects are limited to mild conditions such as visual fatigue and headache [54, 55]. Second, it does not require professional oversight and is easy to administer, with no specific requirements on the environment. Third, patients can engage in other activities, such as reading or listening to music, while receiving light therapy, which enhances treatment adherence. Finally, light therapy is cost‐effective, making it suitable for large‐scale adoption. Taken together, compared to cognitive training, light therapy offers significant advantages and holds considerable value for clinical applications in addiction treatment.

We did not observe significant improvements in compulsive symptoms, emotional symptoms, and sleep quality after light therapy. Compulsive symptoms, which include obsessive thoughts and compulsive behaviors, are one of the frequently observed symptoms in IGD. A recent study developed a 6‐day training protocol targeting compulsive symptoms among IGD [46]. At the end of the intervention, no immediate improvement was observed in the obsessive thoughts or compulsive behaviors of the subjects. Instead, a gradual decreasing trend emerged at 3‐week follow‐up [46]. This suggests that compulsive symptoms may respond to short‐term interventions with some delay. Regarding emotional symptoms, we observed a decreasing trend in depressive and anxiety symptoms, although it did not reach statistical significance. This result is consistent with our earlier findings on light therapy for subthreshold depression individuals [17, 56], which demonstrated significant improvement in depressive and anxiety symptoms after 4‐week light therapy. It is also noted that most clinical trials investigating light therapy for depression typically employ an intervention period of 4 weeks or longer [57]. These findings suggest that light therapy may require a sufficient intervention duration to accumulate its neuromodulatory effects on emotion. Existing evidence has indicated a relationship between internet addiction and reduced sleep quality [58]. The current study used PSQI to measure sleep quality, and we found no significant improvements in PSQI following light therapy. Notably, the participants had an average PSQI score of 7.07 at baseline, suggesting mild sleep difficulties but not severe sleep disturbances. The lack of significant improvement following light therapy may be attributed to the fact that sleep quality was not severely compromised prior to the intervention.

The current results have several clinical implications. First, our current results indicated that light therapy significantly reduced addiction severity, attenuated abstinence‐induced withdrawal and craving symptoms, and decreased weekly gaming time, supporting its efficacy as an effective therapeutic option for IGD. Given the shared neurobehavioral mechanisms between IGD and other types of addiction, light therapy may be a potentially effective intervention for addictive disorders broadly, though this notion requires further investigation. Second, compared with existing intervention approaches for IGD, light therapy offers many advantages, such as noninvasive nature with little adverse effects, convenience for administration, and cost‐effectiveness in both time and financial resources. All these features would promote widespread usage of light therapy in diverse clinical, community and home settings. Finally, the neuroimaging results highlight the potential of habenula, VTA, and OFC as targets for neuromodulation‐based addiction interventions.

Several limitations should be considered. First, follow‐up assessments were conducted only for IGD severity, without evaluating other behavioral and neuroimaging outcomes. Including follow‐up assessments across all outcomes would provide valuable insights into the long‐term effects associated with light therapy. Second, the fMRI study focused exclusively on a cue‐induced craving task design. Investigating brain responses in other mental states, such as during resting‐state or decision‐making tasks, could provide a more comprehensive understanding for the neural mechanisms underlying the effects of light therapy. Finally, although participants were instructed to remain abstinent for 24 h, adherence to this requirement could not be fully verified, introducing a potential confound.

## 4 Conclusions

Light therapy has been shown to effectively reduce addiction severity, weekly gaming time, withdrawal symptoms, and cravings in individuals with IGD. At the neuronal level, light therapy decreased brain activations at the insula, OFC, and IFG, as well as increased functional connectivity of habenula‐mOFC and VTA‐mOFC. The changes in functional connectivity correlated with changes in withdrawal or craving symptoms in the light therapy group. Importantly, light therapy appears to alleviate addiction and withdrawal symptoms by modulating habenula‐mOFC functional connectivity. These findings support the potential of light therapy as a treatment for IGD and offer insights into the neural mechanisms underlying its efficacy.

## 5 Methods Section

### 5.1 Design

**FIGURE 5:**
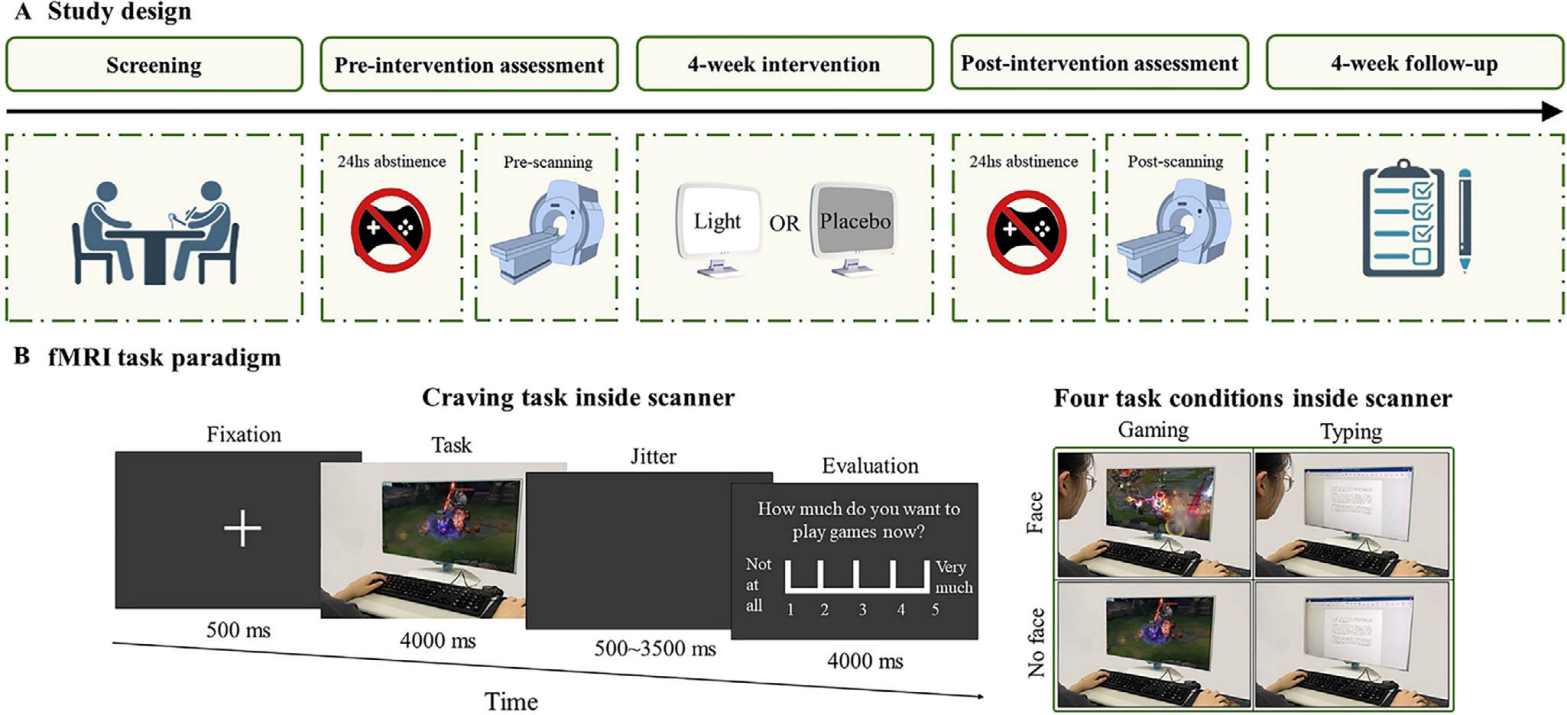
Study design and fMRI task. (A) Study design. (B) Illustration of a trial of the craving task during fMRI. Abbreviations: fMRI, functional magnetic resonance imaging.

The study was preregistered on ClinicalTrials.gov (NCT06165549) and conducted between December 2023 and June 2024 at two universities at in Guangzhou China: Jinan University and South China Normal University. The protocol can be found in Supporting Information. The study was approved by the ethics committee at both universities (JNUKY‐2023‐0045, SCNU‐BRR‐2023‐049) and was performed in accordance with the principles of *Declaration of Helsinki*. This study consisted of two parts (Figure 5). First, an RCT with three groups (light therapy, light placebo, or cognitive training) was conducted to evaluate the efficacy of light therapy for IGD. Cognitive training was selected as an active comparator control group, which served as a benchmark against which to compare the response produced by the light therapy. We also included the light placebo as the sham control group, which created an experience and sensation similar to light therapy by using a light device without an active component of the intervention. By employing two different types of control groups, we are able to comprehensively evaluate the efficacy of light therapy. Second, the participants in the light therapy and light placebo groups were also invited to participate fMRI scans to investigate the neural mechanism underlying light therapy. The fMRI data for the cognitive training group were omitted for two primary reasons. First, our fMRI paradigm requires hypothesis‐driven task selection specifically tailored to the mechanistic framework of light therapy. Second, the neural correlates of cognitive training have already been extensively examined in prior research [46]. The outcome assessments and fMRI scans were performed at baseline and again at the end of the intervention, with the primary outcome also performed at a 4‐week follow‐up. To induce a craving status, participants were requested to abstain from internet gaming for 24 hours before attending outcome assessments. The outcome assessments were conducted in the School of Medicine at Jinan University, and fMRI assessments were conducted in neuroimaging center at South China Normal University.

### 5.2 Participants

Participants were university students recruited through online flyers from Jinan University and South China Normal University. The inclusion criteria were as follows: (1) between 18 and 28 years of age; (2) being right‐handed with normal or corrected‐to‐normal vision; (3) meeting the DSM‐5 criteria for IGD;[2] (4) scoring 50 or higher on the IAT;[59] and (5) playing games for more than 15 h per week for at least one year. Exclusion criteria included: (1) self‐reported current or previous experience with gambling or illegal substances; (2) alcohol use disorder or nicotine dependence; (3) self‐reported history of neurological or psychiatric disorders; (4) current use of psychotropic medications; (5) had photosensitivity disorders, such as systemic lupus erythematosus or chronic actinic dermatitis; and (6) had any MRI contraindications. All participants provided written informed consent and received financial reimbursement (CN ¥280).

### 5.3 Sample Size Estimation

A priori power analysis was conducted using G* Power 3.1.9.6 for a repeated‐measures ANOVA design (3 groups × 3 measurement points) [60]. We refer to existing intervention studies in IGD that used IAT as the primary outcome measure, where effect sizes ranging from 0.2 to 0.4 have been reported in a meta‐analysis study [61]. Since light therapy represents a novel intervention in the field of IGD research, a conservative effect size of 0.2 was selected along with a relatively high power level of 0.95 to minimize the risk of Type II error. With a two‐tailed alpha of 0.05 and a correlation among repeated measures of 0.5, the analysis indicated that 27 participants per group would be required. To account for an anticipated attrition rate of 20%, we aimed to enroll at least 34 IGD participants per group for the RCT study. Our final sample size of 34 participants per group is also consistent with recommendations for statistical power in clinical trials [62].

As there had been no fMRI studies of light therapy in IGD individuals, the sample size for the fMRI component (*n* = 34 per group) was exploratory and aligned with that of the RCT study. The sample size was comparable to those suggested by neuroimaging studies. The guidelines suggest leveraging behavioral outcomes to determine sample sizes in neuroimaging study. This strategy reduces the overall trial burden and facilitates the concurrent investigation of neural mechanisms [63]. The allocation was concealed at the point of recruitment using a computerized random‐number generator in blocks of three. An independent research assistant did the participant allocation, supervised the cognitive training, distributed light boxes, and delivered instructions on the usage of the light box. Study investigators responsible for fMRI scanning and outcome assessment were blind to the group assignment.

### 5.4 Interventions

Eligible participants were randomly allocated (ratio: 1:1:1) to one of the three groups: light therapy, light placebo, or cognitive training group. The intervention consisted of 14 daily 30‐min sessions over two weeks. Following the intervention, we administered a satisfaction survey. Participants in the light therapy and light placebo groups were provided with custom‐made light devices previously utilized in our studies [17, 56]. These devices measured 600 × 300 × 15 mm^3^ with small white light‐emitting diodes (LEDs) inside, 5000‐Kelvin color temperature, and 100% ultraviolet filter. Light intensity was set to 5000‐lux for the light therapy and less than 200‐lux for the light placebo group. All participants received standardized instructions as follows: (a) fully expose the face to the light device; (b) keep the eyes open without staring directly at the light device; (c) position the device on a desk at a distance of 50‐cm from the face. To ensure adherence, the light boxes were equipped with Internet of Things technology to monitor usage (on/off status) in real time. If a device was not activated during a scheduled session, researchers promptly followed up with a phone call to remind the participant. Participants in the cognitive training group received online emotional bias modification training, adapted from a previous study [46].

Participants in the cognitive training group received a 2‐week, daily online training program aimed at regulating gaming behavior. During the training period, investigators monitored progress in real time and provided guidance as needed. The objective of training was to establish associations between gaming cues and negative emotions, thereby reducing compulsive gaming thoughts and behaviors. The training protocol was adapted from a previous study [48]. During the training sessions, gaming‐ and healthy‐activity images were paired with negative and positive emotional word‐pairs, respectively. Participants were requested to select a word from the word‐pair that matched the displayed image. Correct choices had an 80% chance of earning monetary reward (CN ¥0.1 Yuan), while incorrect choices had a 20% chance of getting no reward. The probabilities were reversed for the other word. Participants were encouraged to maximize their monetary rewards, although the reward rules were not explicitly disclosed. At the end of the training, participants received the accumulated monetary rewards they had obtained. Each training trial began with a fixation cross displayed for 600–800 ms, followed by an image and an emotional word pair. Participants had a limited time to respond, followed by feedback displayed for 800 ms. The response time limit progressively decreased over the 14‐day training period: 2000 ms on days 1–3, 1000 ms on days 4–6, 900 ms on days 7–9, 800 ms on days 10–12, and 700 ms on days 13–14. Each daily training session comprised 400 trials (200 gaming‐ and 200 healthy‐activity images), presented in a pseudo‐randomized order. The training utilized 10 gaming‐related images and 10 healthy‐activity images, along with 5 pairs of negative words and 5 pairs of positive words. The gaming images were screenshots from popular games, while healthy‐activity images depicted activities such as playing badminton and basketball, running, skateboarding, reading, singing, and dancing. The images were sourced from pixabay.com. All images were normalized in terms of their overall luminance. Word stimuli were selected from the Chinese Affective Word System [64], and the levels of valence, arousal, and familiarity were matched for the negative (mean valence = 2.81±0.20, mean arousal = 6.23±0.45; mean dominance = 2.68±0.15) and positive words (mean valence = 7.27±0.32; mean arousal = 6.49±0.47; mean dominance = 7.26±0.25).

### 5.5 Outcome Assessment

The primary outcome was predefined as the change in the severity of addiction symptoms over time, measured using the IAT at baseline, postintervention, and 4‐week follow‐up. We present compelling rationale for selecting the IAT as the primary outcome to evaluate IGD severity, as detailed below: (1) from the diagnostic criteria perspective, the IAT aligns well with DSM‐5 and ICD‐11 criteria; (2) in terms of psychometric properties, the IAT shows good reliability and validity in Chinese populations [65]. The Cronbach's α was 0.86 in the current sample. Furthermore, the 4‐week follow‐up assessment was selected as the primary endpoint based on previous studies demonstrating a steady decline in IGD severity throughout this period following the intervention [48, 66].

The secondary outcomes included changes from baseline to postintervention in the following measures: weekly gaming duration, recorded through game history or game‐tracking software; withdrawal symptoms, assessed by the Mood and Physical Symptoms Scale (MPSS);[67] craving, assessed by the total score of two items of MPSS (“how much of the time have you felt the urge to play games today?” and “how strong have the urges been today?”); emotional regulation, assessed by the Emotion Regulation Questionnaire (ERQ);[68] sleep quality, assessed by the Pittsburgh Sleep Quality Index scale (PSQI);[69] obsessive and compulsive thoughts and behaviors, assessed by the Yale–Brown Obsessive–Compulsive Scale modified for IGD (IGD‐YBOCS);[70] depressive and anxiety symptoms assessed by the Beck Depression Inventory [71] and the Beck Anxiety Inventory [72].

Following the intervention, we administered a satisfaction survey to the participants in the light therapy and light placebo groups. The questionnaire comprised five self‐rating items on: overall experience with light intervention using 10‐point scale ranging from “extremely negative” to “extremely positive”; global satisfaction with light intervention using 5‐point scale ranging from “very dissatisfied” to “very satisfied”; willingness to recommend light therapy to peers using binary yes/no response; perceived adequacy of intervention duration using binary yes/no assessment; convenience to use light device using 5‐point scale ranging from “very inconvenient” to “very convenient.”

### 5.6 Neuroimaging Assessment

Imaging data were collected at the Brain Imaging Center of South China Normal University using a 3.0‐Tesla Siemens Prisma Fit MRI system equipped with a 32‐channel phased‐array head/neck coil. Functional images were obtained using a multi‐band gradient‐echo echo‐planar imaging sequence with the standard parameters: repetition time (TR) = 2000 ms, echo time (TE) = 31 ms, flip angle (FA) = 70°, acceleration factor = 2 without GRAPPA, field of view (FOV) = 211 mm × 211 mm, data matrix = 112 × 112, slice thickness = 2 mm with no inter‐slice gap, voxel size = 2 mm^3^, anterior‐to‐posterior phase encoding direction (A> > P), and 62 interleaved slices covering the whole brain. In addition, high‐resolution structural images were acquired using a T1‐weighted 3D MP‐RAGE sequence with the following parameters: TR = 1800 ms, TE = 2.07 ms, FA = 9°, slice thickness = 1 mm, FOV = 256 mm × 256 mm, data matrix = 224 × 224, voxel size = 1 mm^3^, and 176 sagittal slices covering the whole brain.

The fMRI task implemented was a craving paradigm [73]. The stimuli consisted of 40 gaming‐related and 40 typing‐related images, featuring either a face or a hand and balancing for complexity/gender. The gaming‐related images were tailored to each participant, featuring their current favorite game, while the typing‐related images displayed random text content that was consistent across all participants. There were two runs, and each run comprised 40 trials. During each trial, a fixation cross was presented for 500 ms, followed by a stimulus requiring a response within 4000 ms. Participants were instructed to indicate the presence of a face in the image by pressing a button. This was followed by a jittered black screen lasting between 500 and 3500 ms. In the subsequent evaluation stage, participants rated craving level on a five‐point scale ranging from “low” to “high” within 4000 ms, and it was terminated by a button press. After another jitter screen lasting between 500 to 3500 ms, the next trial started.

### 5.7 Data Analyses

Participants who completed all assessments and interventions were included for statistical analyses. Demographics and baseline characteristics were compared across the three groups using one‐way analysis of variance (ANOVA) for continuous variables and Chi‐square/Fisher's exact test for categorical variables. To investigate the intervention effect, the outcomes were analyzed using a linear mixed‐effects model implemented with the *nlme* package in R (https://svn.r‐project.org/R‐packages/trunk/nlme/). The model included group (light therapy, light placebo, and cognitive training) as a between‐subject factor and time (pre‐intervention, postintervention) as a within‐subject factor, with age and gender included as covariates. A random intercept was modeled for each participant. Post hoc analyses were performed for further examination. All analyses were performed using SPSS 22.0 (Armonk, NY, United States) and R (version 4.4.1). A *p *< 0.05 was considered statistically significant, and all significance tests were two‐tailed.

Preprocessing of neuroimaging data included slice timing, realignment of functional time series for head motion correction, co‐registration of functional and anatomical images, segmentation for extracting gray matter, spatial normalization to the Montreal Neurological Institute (MNI) space, and spatial smoothing with a 6‐mm Gaussian kernel. The preprocessed data were fitted to a GLM by convolving the hemodynamic response with a boxcar function. In first‐level analyses, a GLM was built with the following regressors: game‐related, typing‐related, and craving‐rating stage. In addition, six motion parameters were included in the model as regressors of no interest. A high‐pass filter with a cutoff of 1/128 Hz was applied to remove low‐frequency drifts. The individual contrast images were used in second‐level random effects models to examine specific effects. In second‐level analyses, conjunction analysis and two‐sample *t*‐tests were initially conducted to determine common and differential neural activations between the light therapy and light placebo groups at baseline. Intervention effects were evaluated using group (light therapy, light placebo) × time (pre‐intervention, post‐intervention) ANOVA at a whole‐brain level.

Considering the crucial roles of the habenula, VTA, and DRN in light therapy and their associations with addictive symptoms [12], ROI analyses were conducted on brain activations and functional connectivity. These three brain regions were selected as seed regions, each defined by a 3‐mm sphere centered on the peak MNI coordinates identified in previous studies: left habenula = (−2.8, −24.2, 2.3) and right habenula = (4.8, −24.1, 2.2);[74, 75, 76] DRN = (0, −28,−12);[77] left VTA = (−2.7, −15.9, −13.9) and right VTA = (4.1, −15.9, −13.9) [78]. The bilateral habenula and bilateral VTA regions were combined into a single seed for the analysis, respectively. Using the three ROIs as seed regions, we conducted repeated‐measures ANOVA analyses and post‐hoc analyses. Functional connectivity was analyzed using a general form of task‐dependent psychophysiological interaction (gPPI) analyses [79]. Physiological activity in the three seed regions was computed in all voxels as the average time series and was deconvolved to estimate neural activity. Next, neural activity was used to generate PPI regressors for each task condition. The individual‐level PPI effect corresponding to each task condition was entered into a second‐level analysis. Light intervention effects on functional connectivity were evaluated using a group (light therapy, light placebo) × time (pre‐intervention, post‐intervention) ANOVA. For significantly increased or decreased functional connectivity in certain brain regions, estimates were extracted from anatomical ROIs, as defined by the automated anatomical labeling (AAL) atlas [80], for further analysis. Pearson correlation analyses were used to examine relationships between changes in neuroimaging results and clinical outcome measures. Then, a mediation analysis was performed using R to test whether withdrawal/craving symptoms mediate the relationship between changes in functional connectivity and addiction severity.

To examine whether whole‐brain reactivity to craving was decreased in the light therapy group, we conducted additional analysis using a craving neuromarker developed in the study by Kober and colleagues [81]. The researchers used machine learning to construct a cross‐validated neuromarker to reliably predict craving. The Neurobiological Craving Signature (NCS) weight map was obtained from the website (https://github.com/canlab/Neuroimaging_Pattern_Masks/tree/master/Multivariate_signature_patterns/2022_Koban_NCS_Craving) and was resampled to match the resolution and dimensions of our fMRI data. A single NCS score was computed for each participant at both baseline and post‐intervention, which was achieved by extracting the whole‐brain activation pattern from the first‐level contrast image (gaming > typing) and calculating its product with the NCS weight map. The NCS score represents craving‐related neural pattern at individual level, with a higher positive score indicating a better match to the craving state [81]. The NCS scores were analyzed using a repeated‐measures ANOVA and post‐hoc paired *t‐*tests. Neuroimaging data were analyzed using Statistical Parametric Mapping (SPM12, http://www.fil.ion.ucl.ac.uk/spm) and CONN toolbox (CONN22a, https://www.nitrc.org/projects/conn/). Statistical maps were thresholded at a voxel‐level of *p *< 0.001 and a cluster‐level FWE correction of *P*_FWE_ < 0.05.

## Author Contributions

Concept and design: J.L., H.H., C.H., X.Z., L.H., C.R., Q.T.; Acquisition, analysis, or interpretation of data: All authors; Drafting of the manuscript: J.L., H.H., C.H., X.Z., L.H., C.R., Q.T.; Revision of the manuscript: All authors; Statistical analysis: J.L., H.H., L.H., C.R., Q.T.; Obtained funding: C.R., Q.T.; Administrative, technical, or material support: All authors; Supervision: L.H., C.R., Q.T.

## Conflicts of Interest

The authors declare no conflicts of interest.

## Supporting information




**Supporting File**: advs73417‐sup‐0001‐SuppMat.docx.

## Data Availability

Data are available when required by contacting taoqian16@jnu. edu.cn.
