# Supplementary material for: Light Therapy Alleviates Addiction‐Related Symptoms and Reshapes Habenula and Midbrain Pathways
Source: Adv Sci (Weinh). 2026 Jan 14;13(13):e14044. doi: 10.1002/advs.202514044 (PMC12955907; doi:10.1002/advs.202514044)
Supplement: Supplementary file 1 — Supporting File: advs73417‐sup‐0001‐SuppMat.docx. [file ADVS-13-e14044-s001.docx]

**Supporting Information**


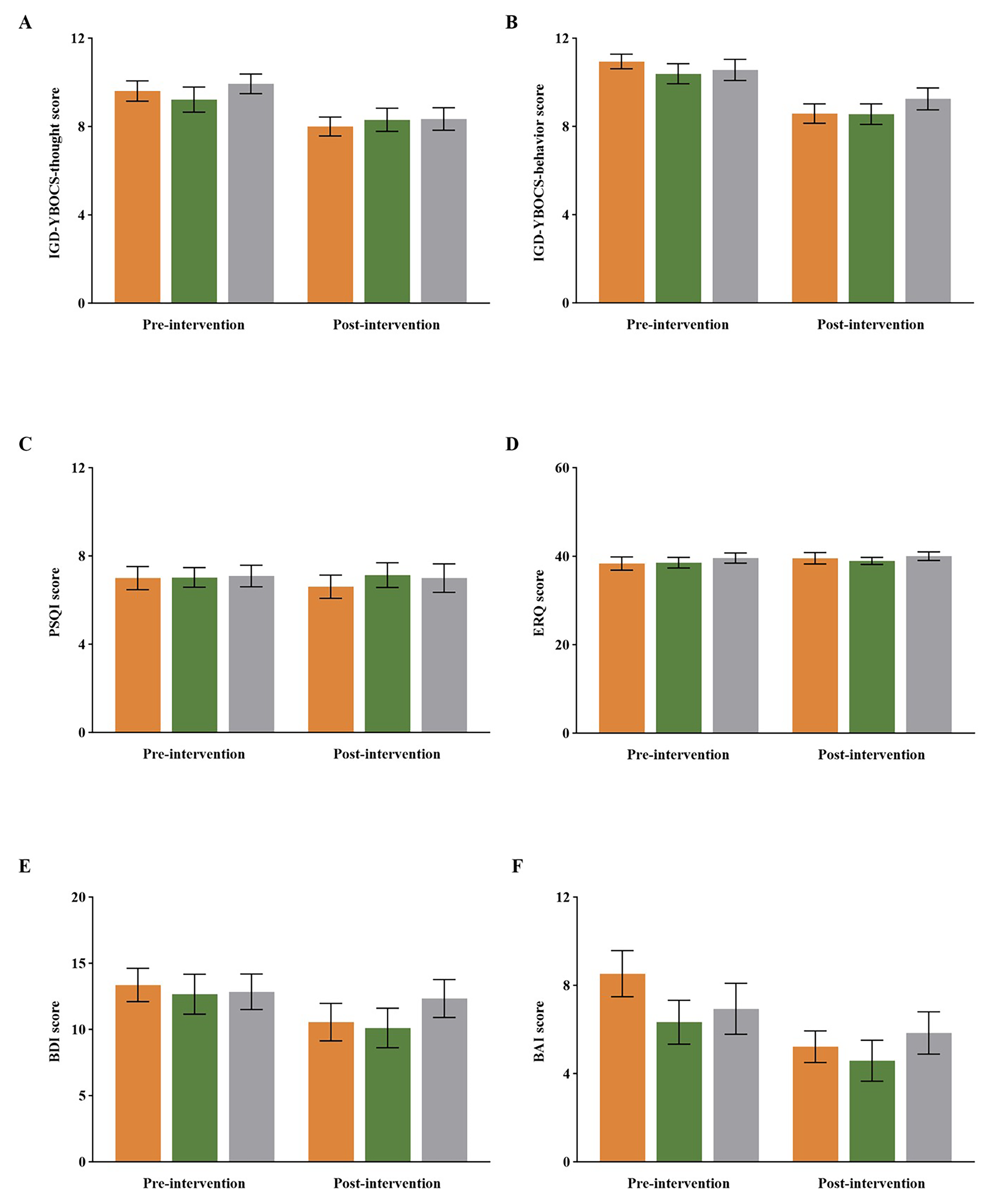


**Figure S1. The effect of intervention on the secondary outcomes.** (A) to (F) shows IGD-YBOCS-behavior scores, IGD-YBOCS-thought scores, PSQI scores, ERQ scores, BDI scores and BAI scores for the light therapy, light placebo and cognitive training groups at pre-intervention and post-intervention. Error bars represent standard errors (SE). Abbreviations: IGD-YBOCS, Yale–Brown Obsessive–Compulsive Scale modified for IGD; PSQI, Pittsburgh Sleep Quality Index; ERQ, Emotion Regulation Questionnaire; BDI, Beck Depression Inventory; BAI, Beck Anxiety Inventory.


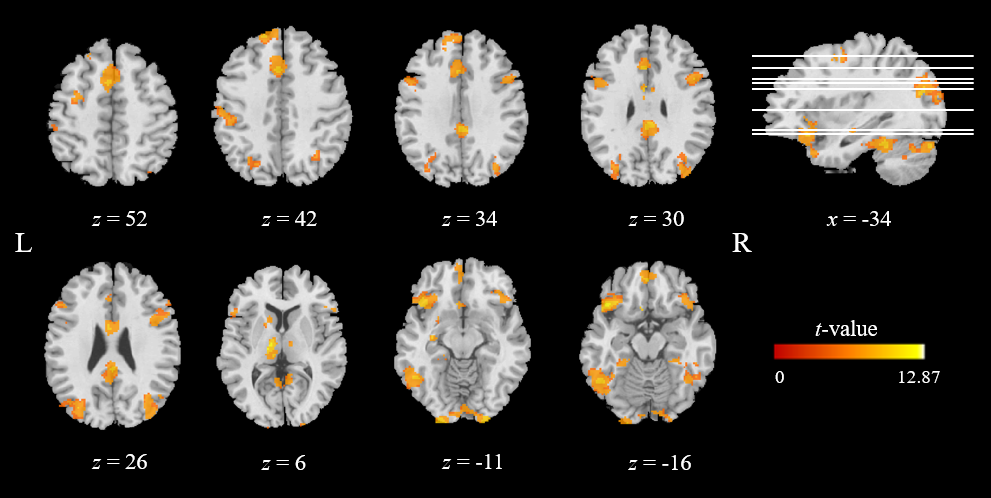


**Figure S2. Brain activations at baseline.** Significant clusters obtained from a conjunction analysis of brain activation during the craving task in the light therapy and light placebo groups at pre-intervention (voxel-level *P* < 0.001 and cluster-level *P*_FWE_ < 0.05). L, left; R, right.

**Table S1.** fMRI results for the conjunction analysis. Common brain regions co-activated during the craving task at the pre-intervention in both light therapy and light placebo groups (voxel-level *P* < 0.001 and cluster-level *P*_FWE_ < 0.05).

| Cluster | L/R | Cluster size (#voxels) | *t* | MNI coordinates | | |
| --- | --- | --- | --- | --- | --- | --- |
|  |  |  |  | *x* | *y* | *z* |
| Thalamus | L | 208 | 11.42 | -15 | -17 | 6 |
| PCC | R | 458 | 10.27 | 3 | -35 | 31 |
| PCC | L | 214 | 9.83 | 0 | -34 | 32 |
| OFC | L | 249 | 9.67 | -39 | 25 | -14 |
| Hippocampus | L | 33 | 8.84 | -31 | -14 | -12 |
| Putamen | L | 189 | 8.23 | -20 | 10 | 1 |
| OFC | R | 169 | 7.88 | 2 | 50 | -20 |
| PreCG | L | 113 | 7.67 | -31 | -2 | 50 |
| IFG | L | 279 | 7.11 | -52 | 16 | 2 |
| ACC | L | 1,106 | 6.88 | -4 | 38 | 16 |
| MTG | L | 832 | 6.81 | -32 | 16 | -34 |
| STG | R | 193 | 6.70 | 44 | 22 | -22 |
| ITG | L | 33 | 6.43 | -62 | -14 | -28 |
| MTG | R | 611 | 6.16 | 52 | -62 | 12 |
| SFG | L | 368 | 6.15 | -18 | 44 | 32 |
| IFG | R | 417 | 6.14 | 58 | 24 | 4 |
| IPL | L | 256 | 6.12 | -45 | -26 | 36 |
| Cerebellum | L | 1,797 | 6.11 | -32 | -62 | -36 |
| MOG | L | 678 | 6.05 | -34 | -88 | 12 |
| Cerebellum | R | 2,720 | 6.04 | 40 | -64 | -44 |

Abbreviations: PCC, posterior cingulate cortex; OFC, orbitofrontal cortex; PreCG, precentral gyrus; IFG, inferior frontal gyrus; ACC, anterior cingulate cortex; MTG, middle temporal gyrus; STG, superior temporal gyrus; ITG, inferior temporal gyrus; SFG, superior frontal gyrus; IPL, inferior parietal lobule; MOG, middle occipital gyrus; FWE, family-wise error; L, left; R, right.

**Table S2.** Intervention effects on whole-brain and ROI activation (voxel-level *P* < 0.001 and cluster-level *P*_FWE_ < 0.05).

| Regions | L/R | Cluster size (#voxels) | *t* | MNI coordinates | | |
| --- | --- | --- | --- | --- | --- | --- |
|  |  |  |  | *x* | *y* | *z* |
| **Whole-brain activation** | | | | | | |
| iOFC | R | 268 | 28.93 | 38 | 22 | -10 |
| Insula | L | 420 | 28.68 | -26 | 24 | -10 |
| IFG | L | 320 | 26.55 | -38 | 34 | 0 |
| MTG | R | 468 | 22.50 | 62 | -28 | -8 |
| mSFG | R | 242 | 26.17 | 2 | 46 | 30 |
| IFG | R | 131 | 25.11 | 40 | 20 | 34 |
| mOFC | R | 177 | 21.20 | 12 | 60 | -12 |
| PreCG | L | 135 | 20.89 | -38 | -2 | 52 |
| Insula | R | 130 | 20.18 | 38 | 28 | -4 |
| **ROI activation** | | | | | | |
| None |  |  |  |  |  |  |

Abbreviations: ROI, region of interest; iOFC, inferior orbitofrontal cortex; IFG, inferior frontal gyrus; MTG, middle temporal gyrus; mSFG, medial superior frontal gyrus; IFG, inferior frontal gyrus; mOFC, medial orbitofrontal cortex; PreCG, precentral gyrus; FWE, family-wise error; L, left; R, right.

**Table S3.** Interaction effects on functional connectivity (voxel-level *P* < 0.001 and cluster-level *P*_FWE_ < 0.05).

| Regions | L/R | Cluster size (#voxels) | *F* | MNI coordinates | | |
| --- | --- | --- | --- | --- | --- | --- |
|  |  |  |  | *x* | *y* | *z* |
| **Seed: habenula** | | | | | | |
| mOFC | L | 76 | > 7.28 | -10 | 54 | -16 |
| ITG | R | 68 | > 7.28 | 60 | -18 | -30 |
| **Seed: VTA** |  |  |  |  |  |  |
| mOFC | R | 71 | > 4.76 | 6 | 54 | 0 |
| **Seed: DRN** |  |  |  |  |  |  |
| Cerebellum | R | 54 | > 7.28 | 30 | -72 | -54 |
| MFG | R | 49 | > 7.28 | 28 | 38 | 42 |
| Precuneus | R | 43 | > 7.28 | 6 | -50 | 58 |

Abbreviations: mOFC, medial orbitofrontal cortex; ITG, inferior temporal gyrus; VTA: ventral tegmental area; DRN: dorsal raphe nuclei; MFG, middle frontal gyrus; FWE, family-wise error; L, left; R, right.
